# Neural and Mechanical Adaptations During Static Stretching With Different Amplitudes

**DOI:** 10.1111/ejn.70475

**Published:** 2026-04-10

**Authors:** Denis César Leite Vieira, Martim Bottaro, Marion Hitier, João Luiz Quaglioti Durigan, Nicolas Babault

**Affiliations:** ^1^ Université Bourgogne Europe INSERM, CAPS UMR 1093 Dijon France; ^2^ College of Physical Education, Strength and Conditioning Research Laboratory University of Brasilia Brasília Brazil; ^3^ Graduate Program of Rehabilitation Sciences, Laboratory of Muscle and Tendon Plasticity University of Brasilia Brasília Brazil

**Keywords:** muscle performance, neural adaptations, peripheral adaptations, stretch, warm‐up

## Abstract

This study examined neural and mechanical responses at the onset of prolonged static stretching performed at different amplitudes, their progression during the stretching period, and alterations immediately after its completion. Thirteen healthy adults completed three randomized sessions: control (15‐min rest in neutral ankle angle), 15 min of submaximal stretching (ROM_max_ − 5°), and 15 min of supramaximal stretching (ROM_max_ + 5°). Spinal excitability (*H*_max_/*M*_max_), evoked contractile properties (PTT), voluntary strength (MVC), passive torque, and maximal range of motion (ROM_max_) were assessed at baseline (PRE), during stretching (T_00_, T_05_, T_10_, and T_15_), and immediately after stretching (POST). Spinal excitability decreased at stretch onset but progressively increased during stretching (T_00_–T_15_, *p* < 0.05), with greater facilitation in supramaximal (soleus and gastrocnemius, *p* < 0.001) than submaximal conditions (soleus only, *p* < 0.001). These changes were transient, returning to baseline at POST (*p* < 0.001). Passive torque increased during both stretching (*p* < 0.05); however, in supramaximal, it declined after 5 min and stabilized until 15 min (*p* = 0.001), while in submaximal, it declined only at 15 min (*p* = 0.043). Both conditions reduced PTT (*p* < 0.05), with slightly greater decreases in the supramaximal condition. MVC decreased similarly after both protocols (*p* < 0.05). ROM_max_ improved in both (*p* < 0.05), with slightly greater gains in the supramaximal condition. Static stretching amplitude modulates neuromechanical adaptations. Both supramaximal and submaximal protocols reduced voluntary strength but improved flexibility, with greater spinal excitability after the initial decrease and slightly larger ROM_max_ gains at supramaximal amplitude.

## Graphical abstract

Prolonged static stretching elicits biphasic modulation of spinal excitability characterized by initial suppression followed by progressive facilitation that is amplitude‐dependent, with supramaximal stretching producing greater neural adaptations in both soleus and gastrocnemius muscles. In contrast, submaximal stretching only affects the soleus. Concurrently, passive torque initially increased during stretch application but subsequently declined more rapidly under supramaximal conditions (5 min) compared to submaximal conditions (15 min), indicating differential viscoelastic adaptations within the muscle‐tendon unit.

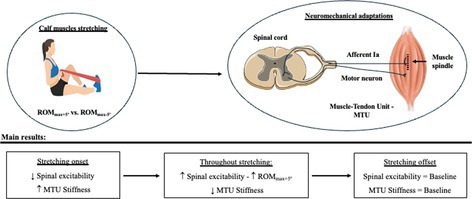

**Abbreviations**
CON: control condition
EMG: electromyography
*E*_reflex_/*M*_max_
: exteroceptive reflex normalized by maximal M wave
ES: effect size
GM: gastrocnemius medial
*H*_max_/*M*_max_
: maximal Hoffman reflex normalized by maximal M wave
*H*_reflex_
: Hoffman reflex
MEP/*M*_max_
: motor evoked potential normalized by maximal M wave
*M*_max_
: maximal M wave
MVC: maximal voluntary contraction
PIC: persistant inward current
POST: post moment measures
PRE: PRE moment measures
PT: passive torque
PTT: peak twitch torque
ROM_max_
: maximal range of motion
Submaximal: submaximal static stretching condition
Supramaximal: supramaximal static stretching condition
T_00_
: measures at the beginning of static stretching
T_05_
: measures 5 min after the beginning of stretching
T_10_
: measures 10 min after the beginning of stretching
T_15_
: measures 15 min after the beginning of stretching
*T*_reflex_
: tendon reflex
*T*_reflex_/*M*_max_
: tendon reflex normalized by maximal M wave

## 1 Introduction

Static stretching has been consistently reported to impair muscle strength and power acutely (Behm and Chaouachi 2011; Behm et al. 2016; Warneke et al. 2025). Despite this evidence, it remains widely employed by physically active individuals, primarily due to its effectiveness in enhancing joint range of motion (ROM) (Babault et al. 2021). Indeed, evidence from the literature consistently demonstrates that static stretching elicits acute increases in joint ROM and flexibility (Behm and Chaouachi 2011; Behm et al. 2016; Warneke et al. 2025). Moreover, these effects appear to be duration‐dependent, with longer stretching protocols producing greater improvements compared to shorter ones (Matsuo et al. 2013). Consequently, prolonged static stretching with varying amplitudes has been widely implemented in clinical practice to improve joint flexibility (Behm et al. 2001; Warneke et al. 2025). However, the influence of stretching amplitude on acute flexibility adaptations following long‐lasting static stretching (i.e., > 5 min) remains uncertain. This uncertainty largely arises because most previous studies have investigated different amplitudes only within shorter protocols (i.e., 20 s to ~3 min), and their results have been inconsistent (Kataura et al. 2017; Santos et al. 2020; Takeuchi, Sato, Kiyono, et al. 2021; Marchetti et al. 2022).

It has been proposed that neural mechanisms, particularly spinal excitability, play a key role in determining muscle flexibility and may thus contribute to adaptations in joint ROM (Guissard et al. 2001). For instance, spinal excitability mediated by afferent pathways conveys input from muscle spindles, that is, proprioceptive receptors arranged in parallel with muscle fibers and innervated by group Ia and II afferent fibers, to spinal motor neurons (Matthews 1931; Trajano and Blazevich 2021). These receptors can mediate stretch reflexes during stretching maneuvers, theoretically increasing motor neuron excitability and muscle activation, thereby influencing ROM assessments (Gollhofer and Rapp 1993; Blazevich et al. 2014; Mizuno 2017). Consequently, most assessments of passive maximal ROM and muscle–tendon stiffness are performed at low angular velocities to minimize stretch reflex activation from muscle spindle afferents, which could otherwise elevate spinal excitability and, consequently, passive tension during muscle lengthening (Blazevich et al. 2014; Bouvier et al. 2017; Mizuno 2017; Vieira et al. 2021, 2025).

However, evidence regarding the effects of static stretching on spinal excitability remains inconsistent (Budini et al. 2019; Pulverenti et al. 2020). For example, studies using the Hoffman reflex normalized to the maximal M wave (*H*_max_/*M*_max_) to control for changes in efferent muscle fiber excitability and/or the tendon reflex (*T*_reflex_) have reported that static stretching can acutely modulate spinal excitability (Budini et al. 2017, 2019). These effects, however, appear to be transient, returning to baseline within minutes after stretching cessation (Budini et al. 2019). Conversely, other investigations assessing the tendon reflex normalized by the maximal M wave (*T*_reflex_/*M*_max_) have found no significant changes in spinal excitability following static stretching (Pulverenti et al. 2021), and protocols involving five 60‐s bouts of static stretching have similarly shown no effect on *H*_max_/*M*_max_ (Pulverenti et al. 2020). These inconsistencies may be partly attributable to methodological differences, including variations in normalization procedures (e.g., normalization to *M*_max_), heterogeneity in stimulation protocols used to assess *H*_max_/*M*_max_, *T*_reflex_, and *T*_reflex_/*M*_max_, as well as differences in stretching volume across the studies (Budini et al. 2017, 2019; Pulverenti et al. 2020, 2021). Beyond these inconsistencies, the acute effects of long‐lasting static stretching performed at amplitudes above or below the maximal point of discomfort, an approach widely implemented in clinical practice to improve joint flexibility, remain unclear (Behm et al. 2001; Warneke et al. 2025).

In addition to neural mechanisms, peripheral adaptations within the muscle–tendon unit are thought to contribute to acute increases in joint ROM (Matsuo et al. 2013; Hirata et al. 2017). The acute mechanical responses of the muscle–tendon unit to prolonged static stretching are relatively well established (Matsuo et al. 2013; Hirata et al. 2016). For instance, reductions in evoked contractile responses have been observed following 20 min of static stretching (Behm et al. 2001), which may reflect alterations in cross‐bridge function and muscle–tendon stiffness (Ryan et al. 2011). Supporting this, both shear‐wave elastography and torque–angle analyses consistently demonstrate that 5 min of stretching performed at maximal tolerable discomfort decreases muscle–tendon stiffness (Matsuo et al. 2013; Hirata et al. 2017). These findings suggest that static stretching can reduce the viscoelastic properties of the muscle–tendon unit, thereby lowering passive tension during muscle lengthening and contributing to improvements in joint ROM. However, as with neural mechanisms, the available evidence is limited to stretching protocols performed up to the maximal point of discomfort (Matsuo et al. 2013; Hirata et al. 2016, 2017); therefore, the influence of different static stretching amplitudes on muscle–tendon stiffness responses remains insufficiently understood.

While most investigations have focused on the acute outcomes observed following static stretching (Hirata et al. 2017; Budini et al. 2019; Pulverenti et al. 2021), the neurophysiological and mechanical adaptations that occur during the stretching maneuver itself remain less well characterized (Guissard et al. 2001; Opplert and Babault 2018). Current evidence suggests that spinal excitability decreases during muscle lengthening (Guissard et al. 2001) and passive static stretching (Budini et al. 2018), with the magnitude of inhibition being proportional to the extent of muscle lengthening throughout the stretching maneuver (Guissard et al. 2001; Guissard and Duchateau 2006). Additionally, muscle–tendon stiffness may transiently increase with muscle lengthening (Mizuno and Umemura 2016; Mizuno 2017). However, these findings are primarily derived from protocols involving short‐duration static stretches and/or single muscle‐lengthening movements (Guissard et al. 2001; Mizuno and Umemura 2016; Mizuno 2017; Budini et al. 2018), which in turn may not represent the most effective stretching protocols to be applied in sports and rehabilitation settings to acutely enhance the ROM (Babault et al. 2021; Warneke et al. 2025). Indeed, as previously reported, despite inconsistent findings concerning stretching amplitude (Kataura et al. 2017; Santos et al. 2020; Takeuchi, Sato, Kiyono, et al. 2021; Marchetti et al. 2022), greater improvements in flexibility have been observed with longer‐duration static stretching protocols (Matsuo et al. 2013).

Therefore, the neurophysiological and mechanical responses elicited by the most adequate protocols for acute ROM improvements, particularly prolonged static stretching performed at varying amplitudes, remain poorly understood. Thus, given the importance of characterizing these responses to optimize the safety and effectiveness of stretching prescriptions (Guissard et al. 2001; Vianna et al. 2009), and considering that the acute effects of long‐lasting static stretching performed at amplitudes above or below the maximal point of discomfort remain unclear. This study was designed to examine neural and mechanical responses at the onset, during, and immediately after prolonged static stretching performed at different amplitudes. We hypothesized that static stretching would transiently reduce spinal excitability, particularly at higher amplitudes, but would progressively increase throughout the stretching period with larger enhancements at higher amplitudes. Conversely, muscle‐tendon stiffness was hypothesized to decrease during stretching, with greater reductions at larger amplitudes.

## 2 Methods

### 2.1 Ethical Approval

The study received approval from the Ethics Committee for Research in STAPS (CERSTAPS IRB00012476‐2023‐17‐02‐229). All individuals were fully informed about the experimental procedure and purpose of the study, and they read and signed an informed consent form. The study followed the standards set out by the *Declaration of Helsinki*.

### 2.2 Participants

Thirteen volunteers (7 women and 6 men) participated in this study. The mean age ± standard deviation (SD), height, body mass, and physical activity per week were 27.2 ± 6.8 years, 170.7 ± 9.7 cm, 69.9 ± 16.9 kg and 5.6 ± 2.8 h. None of the participants reported any lower limb injuries or back pain in the last 3 months, nor any specific hamstrings or triceps surae injuries in the previous 2 years. During the present experiment, participants were asked to maintain their regular physical activities and dietary intake. They were also advised to refrain from intensive activity for at least 2 days before an experimental session.

### 2.3 Experimental Procedures

A randomized, controlled, and crossover design was used to explore acute neuromechanical adaptations during and immediately after 15 min of static stretching performed at different amplitudes. Participants visited our lab on four occasions separated by 72 h. The first visit was used to familiarize volunteers with all test procedures and static stretching protocols. The following three other experimental sessions (randomly presented) were: (1) control session (control); (2) 15 min of static stretching performed at supramaximal amplitudes (supramaximal); and (3) 15 min of static stretching performed at submaximal amplitudes (submaximal).

**FIGURE 1:**
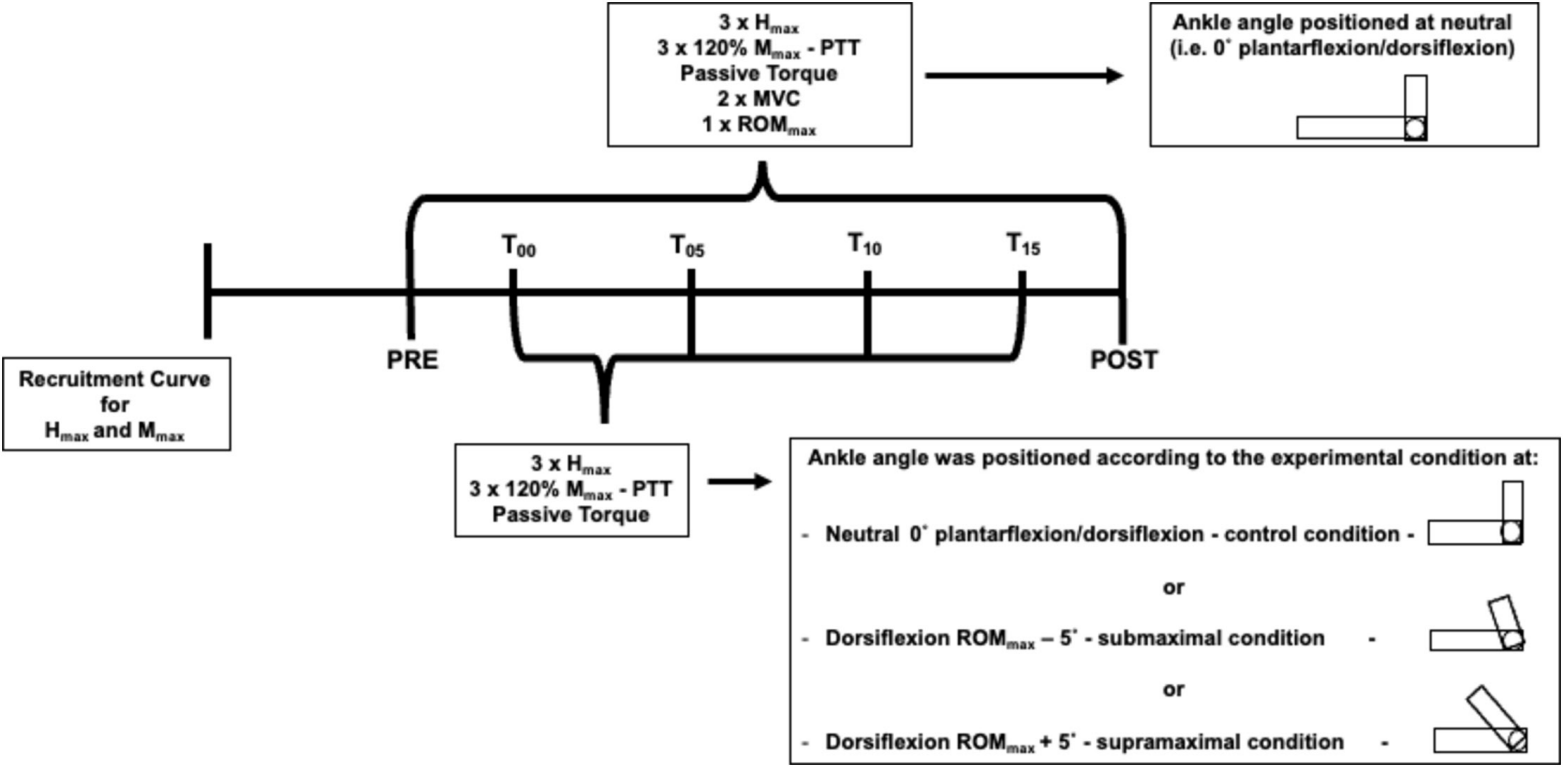
Experimental design. Control = experimental protocol with ankle positioned at neutral, that is, no stretching, *H*_max_ = maximal Hoffman reflex, *M*_max_ = maximal *M*_wave_, MVC = maximal voluntary contraction, POST = measurements immediately after experimental protocol, PRE: measurements before the experimental protocol, ROM_max_ = maximal range of motion, Submaximal = experimental protocol with ankle position during the stretching at ROM_max_ − 5°, Supramaximal = experimental protocol with ankle position during the stretching at ROM_max_ + 5°, T_00_ = measurement at the onset of experimental protocol, T_05_ = measurement 5 min after the onset of experimental protocol, T_10_ = measurement 10 min after the onset of experimental protocol, T_15_ = measurement 15 min after the onset of experimental protocol.

During all experimental sessions, no warm‐up was performed to avoid any influence on pretests and to verify the effect of static stretching as an isolated warm‐up component. Initially, the recruitment curves from tibial nerve electrical stimulation were performed to identify the intensities relative to *H*_max_ and *M*_max_. Subsequently, participants remained at rest for 10 min. After this rest period, the pre‐tests (PRE) were conducted, with the ankle at neutral position (i.e., 0°), to determine spinal excitability (*H*_max_/*M*_max_), muscle performance (maximal isometric voluntary contraction [MVC] and maximal range of motion [ROM_max_]), and muscle‐tendon stiffness (passive torque [PT] and peak twitch torque). These test procedures were repeated immediately after (POST) each experimental condition (Figure 1).

Additionally, during the experimental protocols, at each 5 min from the begin of the protocol (T_00_, T_05_, T_10_, and T_15_), six tibial nerve electrical stimulation were done at intensities relative to *H*_max_ and *M*_max_ (three electrical stimulation at each intensity), and PT were register to measure the dynamics of changes in spinal excitability, muscle‐evoked contractile properties, and muscle‐tendon stiffness during prolonged static stretching performed at different amplitudes and control conditions. The PRE and POST measures were done with ankle at neutral position, that is, 0° of plantarflexion/dorsiflexion in all experimental conditions, and T_00_, T_05_, T_10_, and T_15_ measures were done with the ankle positioned according to the daily experimental protocol, that is, supramaximal (ROM_max_ + 5°), submaximal (ROM_max_ − 5°), or control (neutral position). Therefore, the experimental protocol was not interrupted to perform the assessment.

All experimental procedures were completed on the right plantar flexor muscles using an isokinetic dynamometer (Biodex System 4, BIODEX Corporation, Shirley, NY, USA). The participants were positioned in the supine position, with the hip in a neutral position (0° flexion/extension), the right knee fully extended, and the left knee flexed at 90° in all experimental sessions. To minimize heel displacements, the foot was positioned and fastened inside a shoe and firmly attached to the footplate of the dynamometer with straps. The lateral malleolus was aligned to the center of rotation of the dynamometer. Volunteers' positioning was carefully registered and reproduced to permit consistent measurements during the different experimental sessions.

### 2.4 Electromyography

Surface electromyography (EMG) activity was recorded from the soleus (SOL) and gastrocnemius medialis (GM) muscles of the right leg. The skin was shaved and cleaned with alcohol to minimize impedance. Bipolar silver–chloride electrodes (10‐mm diameter, 2‐cm interelectrode distance) were used. For SOL, electrodes were placed 2 cm distal to the Achilles tendon intersection, while for GM, they were positioned over the muscle belly. The reference electrode was placed over the contralateral patella (Pulverenti et al. 2020). The EMG signal was amplified with a bandwidth frequency range from 10 Hz to 5 kHz (gain = 500). EMG traces were recorded using a Biopac MP 200 system (Biopac System, Santa Barbara, CA, USA) at a sampling rate of 10 kHz and stored for analysis with the AcqKnowledge software (Ver. 6.0, Biopac System, Santa Barbara, CA, USA).

### 2.5 H‐Reflex, M‐Wave, Peak Twitch Torque, and PT Assessments

Transcutaneous electrical nerve stimulation was used to assess the H‐reflex, M‐wave, and Peak Twitch Torque (Opplert and Babault 2019; Vieira et al. 2021, 2025). Additionally, PT assessment was performed immediately prior to the transcutaneous electrical nerve stimulation (Vieira et al. 2025). The posterior tibial nerve was stimulated with rectangular pulses (1 ms) using a Digitimer stimulator (DS7R, Hertfordshire, UK). The cathode electrode (10 mm in diameter) was placed in the popliteal fossa, and the anode electrode (5 × 10 cm) was placed over the patella on the anterior surface of the knee. The optimal stimulation site was determined by eliciting the largest M‐wave at a given intensity using a handheld stimulation probe. Then, the stimulation electrode was pasted to the site (Vieira et al. 2025).

**FIGURE 2:**
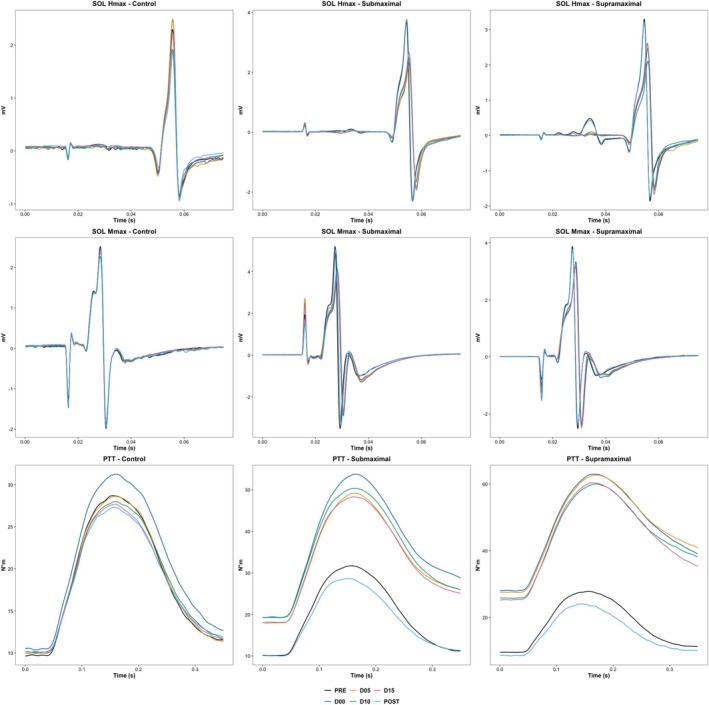
Representative waveforms from raw data of one participant (i.e., same individual in all time points of all experimental conditions). Control = experimental protocol with ankle positioned at neutral, that is, no stretching, *H*_max_ = maximal Hoffman reflex, *M*_max_ = maximal *M*_wave_, mV = millivolts, N*m = Newton/m, POST = measurements immediately after experimental protocol, PRE: measurements before the experimental protocol, Submaximal = experimental protocol with ankle position during the stretching at ROM_max_ − 5°, Supramaximal = experimental protocol with ankle position during the stretching at ROM_max_ + 5°, T_00_ = measurement at the onset of experimental protocol, T_05_ = measurement 5 min after the onset of experimental protocol, T_10_ = measurement 10 min after the onset of experimental protocol, T_15_ = measurement 15min after the onset of experimental protocol.

The recruitment curve was obtained by increasing stimulation intensity in 2‐mA increments from 0 mA to the SOL maximal peak‐to‐peak H‐reflex amplitude (*H*_max_). After reaching *H*_max_, 5‐mA increments were used until the SOL maximal peak‐to‐peak M‐wave amplitude (*M*_max_). For PRE and POST measures, as well as for measures during experimental protocols (T_00_, T_05_, T_10_, and T_15_), three electrical stimuli were delivered at an intensity relative to the SOL *H*_max_, and three electrical stimuli at 120% of the SOL *M*_max_ were delivered, with a 10‐s rest interval (Figure 2). The intensities were the same for PRE, T_00_, T_05_, T_10_, T_15_, and POST moments of each experimental session. The use of a constant current intensity throughout an experimental session is common in the literature (Opplert et al. 2020; Vieira et al. 2025). The order of stimuli (i.e., *H*_max_ or *M*_max_) was randomized for each participant. The mean H‐reflex and M‐wave amplitudes of the SOL and GM, obtained from the three stimuli at each intensity relative to the SOL *H*_max_ and SOL *M*_max_, were considered the *H*_max_ and *M*_max_ of each muscle. *H*_max_ was then normalized by *M*_max_ (*H*_max_/*M*_max_) to minimize the influence of peripheral changes. Additionally, the torque was recorded for the three electrical stimuli delivered at 120% of *M*_max_, and the mean peak torque induced by plantar flexion across the three attempts was used for PTT analysis (Opplert and Babault 2019; Vieira et al. 2021, 2025).

Moreover, for PT analysis, ankle torque was recorded from a 10‐millisecond window immediately preceding the first electrical stimulation at each time point (i.e., PRE, T_00_, T_05_, T_10_, T_15_, and POST) in each experimental condition (Vieira et al. 2025). PT is often considered a marker of muscle–tendon stiffness, and evoked contractile properties (i.e., peak twitch torque), although primarily reflecting contractile capacity, may also indicate changes in muscle and tendon stiffness. Therefore, both PT and peak twitch torque were considered as surrogate markers of muscle‐tendon stiffness for our purpose (Ryan et al. 2011; Vieira et al. 2021).

### 2.6 Maximal Voluntary Contraction

Two isometric plantar flexor Maximal voluntary contractions (MVCs) with the ankle at a neutral position were performed, respecting a rest interval of 1 min to determine the maximal voluntary peak torque. The individuals were verbally encouraged to produce maximal force during MVCs; the peak torque for each MVC was recorded, and the highest value was used for analysis.

### 2.7 Range of Motion

ROM_max_ was assessed using an isokinetic dynamometer (Biodex System 4, BIODEX Corporation, Shirley, NY, USA). Participants were positioned supine, with the hip in neutral (0° flexion/extension), the right knee fully extended, and the left knee flexed to 90°. During the ROM_max_ assessment, the ankle was passively dorsiflexed by the dynamometer at a constant angular velocity of 5°·s^−1^, starting from the neutral ankle position (0° plantarflexion/dorsiflexion). Participants were instructed to press a handheld button to voluntarily terminate the movement at the point of maximal stretch discomfort. ROM_max_ was defined as the ankle dorsiflexion angle at which the participant pressed the button to stop the movement (Vieira et al. 2025).

### 2.8 Experimental Protocols

During static stretching, participants lay supine with the hip in a neutral position (0° flexion/extension), the left knee flexed at 90°, the right knee fully extended, and the right foot secured to the isokinetic dynamometer. In both stretching conditions, the ankle was passively rotated at a slow velocity (5°·s^−1^) from the neutral position to either 5° beyond (supramaximal) or 5° short of (submaximal) the previously determined maximal passive dorsiflexion at the beginning of each experimental session. Participants then remained in the stretched position for 15 min. During the control session (CON), participants rested in the supine position for 15 min with the ankle secured to the dynamometer in the neutral position.

### 2.9 Statistical Analysis

The data were reported as mean ± standard deviation (SD). Data reliability was assessed using the intraclass correlation coefficient (ICC_3,3_), with ICC scores < 0.5 considered poor, 0.5–0.74 moderate, 0.75–0.9 good, and > 0.9 excellent (Atkinson and Nevill 1998; Koo and Li 2016). Moreover, a generalized mixed model with preintervention measurements for each condition (supramaximal, submaximal, and control) as fixed factors, and participants as a random intercept to account for interindividual variability, was also used to assess systematic error (Atkinson and Nevill 1998). The main aim of the study was assessed using the generalized mixed model analysis with condition (supramaximal, submaximal, and control), and time (PRE, T_00_, T_05_, T_10_, T_15_, and POST for SOL and GM *H*_max_/*M*_max_, PTT and PT; and PRE, and POST for MVC and ROM_max_) as fixed factors, and participants as random intercept to account for interindividual variability. In addition, for SOL and GM *H*_max_/*M*_max_, PTT, and PT, the T_00_ values were included as covariates. Additionally, only for PTT, the PT of all moments (i.e., PRE, T_00_, T_05_, T_10_, T_15_, and POST) were also included as covariates, since all experimental protocols were performed with the ankle in different positions, which could affect both *H*_max_/*M*_max_, PTT, and PT measures (Guissard et al. 2001). When a significant main effect or interaction was detected, a Tukey post hoc test was conducted. The significance level for all comparisons was set at *p* < 0.05. The effect size between PRE and POST, and between T_00_ and T_15_ measures were calculated from the following formulas: POST mean − PRE mean/PRE SD, T_15_ mean − T_00_ mean/T_00_ SD, respectively, and classified as trivial (< 0.35), small (0.35–0.80), moderate (0.80–1.50), and large (> 1.5) effect size (Rhea 2004). All statistical procedures were performed using Jamovi Software (Version 2.6.44, The Jamovi Project, Sydney, Australia, available free at http://jamovi.org). Additionally, sample size power was determined a posteriori using RStudio (Ver 2023.03.1, Posit Software, PBC, Boston, MA, USA), following the recommendations of Kumle et al. (2021) for power analyses with the available data as the starting point. We defined the SOL *H*_max_/*M*_max_ as the primary dependent variable. Our sample of 13 subjects was used as a random intercept. The subjects were assessed at 6 time points (PRE, T_00_, T_05_, T_10_, T_15_, and POST) across three conditions (control, submaximal, and supramaximal), with time and condition treated as fixed factors of the generalized mixed model. Therefore, we had 234 measures for the primary variable. Thus, considering the procedures of Kumle et al. (2021) for power analyses with the available data as the starting point, the power was 0.89 and 0.94 for 12 and 13 subjects, respectively. Given that a power > 0.80 is considered good to excellent (Kumle et al. 2021), we deemed that the sample size of 12–13 subjects was sufficient. The dataset, custom RStudio code for power analyses, and figures showing individual data points are available at https://github.com/denisclvieira/STRETCHAMPLITUDE.git.

## 3 Results

There were no significant differences between the pre‐intervention conditions for SOL *H*_max_/*M*_max_ (*p* = 0.362), GM *H*_max_/*M*_max_ (*p* = 0.409), PTT (*p* = 0.196), PT (*p* = 0.072), MVC (*p* = 0.349), and ROM_max_ (*p* = 0.125), reporting no systematic error between the measures. Additionally, data reliability was good to excellent, as ICC_3,3_ values were 0.848 (good), 0.780 (good), 0.847 (good), 0.941 (excellent), 0.980 (excellent), and 0.973 (excellent) for SOL *H*_max_/*M*_max_, GM *H*_max_/*M*_max_, PTT, PT, MVC, and ROM_max_, respectively.

**FIGURE 3:**
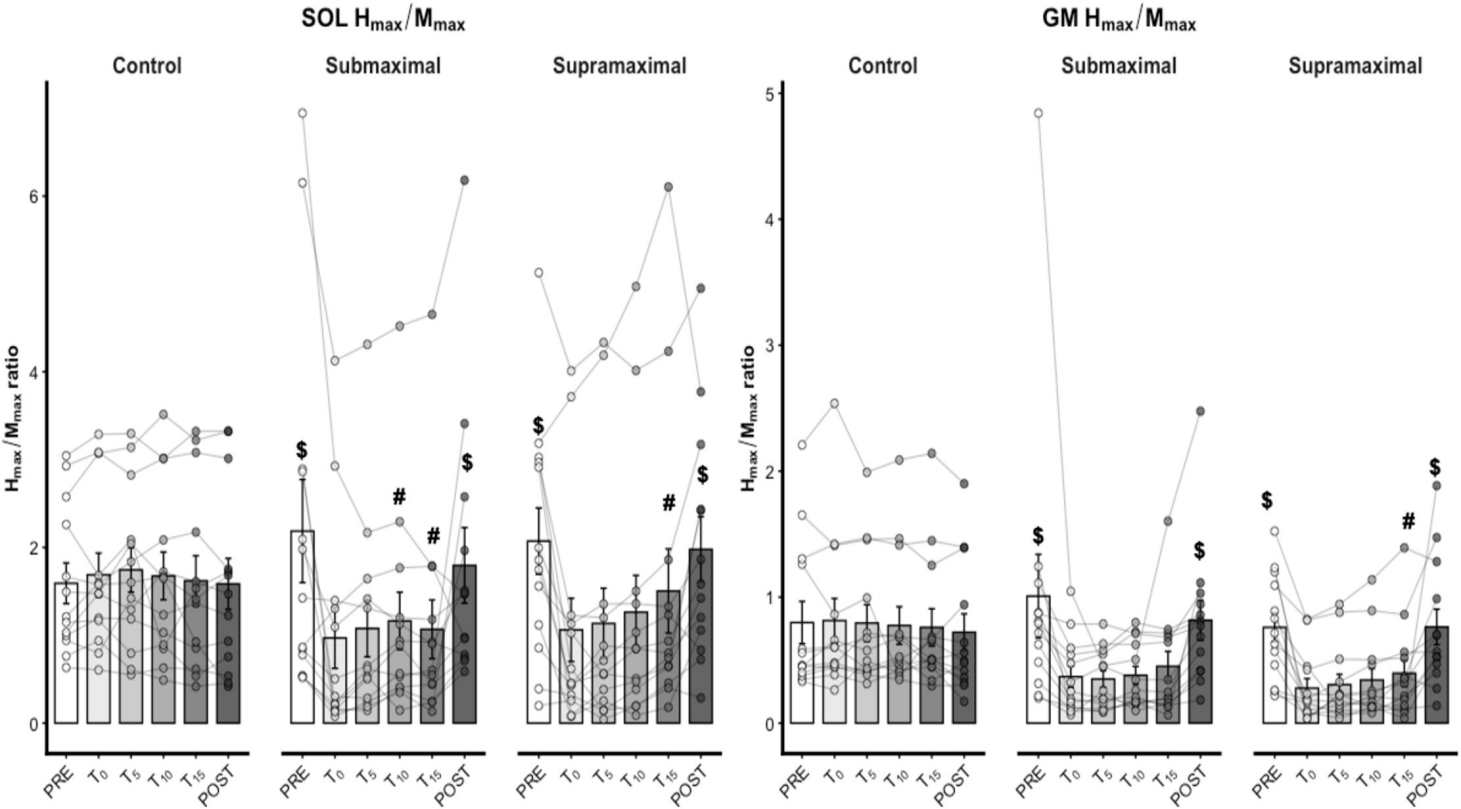
Neural adaptations (*n* = 13 participants). Control = control session, ES _PREvs.POST_ = effect size between PRE and POST moments, ES _T00vs.T15_ = effect size between T_00_ and T_15_ moments, GM *H*_max_/*M*_max_ = gastrocnemius medial maximal *H*_reflex_ normalized by maximal *M*_wave_, POST = posttest, PRE = pretest, SOL *H*_max_/*M*_max_ = soleus maximal *H*_reflex_ normalized by maximal *M*_wave_, Submaximal = static stretching submaximal, Supramaximal = static stretching supramaximal, T_00_ = measure immediately at the begin of the experimental condition, T_05_ = measure 5 min after the begin of the experimental condition, T_10_ = measure 10 min after the begin of the experimental condition, T_15_ = measure 15 min after the begin of the experimental condition. ^#^Significant differences with the T_00_ (*p* < 0.05). ^$^Significant differences with T_00_, T_05_, T_10_, and T_15_ (*p* < 0.01).

For SOL *H*_max_/*M*_max_, there was no significant effect of condition (χ^2^ = 1.14, *p* = 0.565). However, there was a significant main effect of time (χ^2^ = 60.40, *p* = 0.001) and a significant time × condition interaction (χ^2^ = 91.03, *p* = 0.001). SOL *H*_max_/*M*_max_ at T_15_ was greater than T_00_ and T_05_ in the supramaximal condition (*p* < 0.05), and T_00_ in the submaximal condition (*p* = 0.013). Additionally, SOL *H*_max_/*M*_max_ at T_10_ was greater than T_00_ in the submaximal condition (*p* = 0.004). SOL *H*_max_/*M*_max_ at T_00_, T_05_, T_10_, and T_15_ were significantly lower than at PRE and POST in both supramaximal (*p* < 0.01) and submaximal (*p* = 0.01) conditions. Conversely, there were no significant differences between PRE and POST SOL *H*_max_/*M*_max_ in any experimental condition (*p* > 0.05), nor between T_00_, T_05_, T_10_, and T_15_, in the control condition (*p* > 0.05, Figure 3, Table 1). SOL *H*_max_/*M*_max_ effect sizes between PRE and POST were 0.05 (trivial), 0.24 (trivial), and 0.26 (trivial) for control, submaximal, and supramaximal conditions, respectively. Additionally, SOL *H*_max_/*M*_max_ effect sizes between T_00_ and T_15_ were −0.02 (trivial), 0.26 (trivial), and 0.42 (small) for control, submaximal, and supramaximal conditions, respectively.

**TABLE 1:** Summary of statistical effects (time, condition, and interaction).

Comparisons/variables		SOL *H*_max_/*M*_max_	GM *H*_max_/*M*_max_	PTT	Passive torque	MVC	ROM_max_
TIME MAIN EFFECT							
PRE vs. POST		↔	↔	↓	↔	↑	↓
T_00_ vs. PRE		↓	↓	↑	↑	—	—
T_00_ vs. T_05_		↔	↔	↔	↑	—	—
T_10_ vs. T_00_		↔	↔	↓	↓	—	—
T_15_ vs. T_00_		↑	↑	↓	↓	—	—
POST vs. T_15_		↑	↑	↓	↓	—	—
CONDITION MAIN EFFECT							
Control vs. submaximal		↔	↔	↔	↓	↓	↓
Control vs. supramaximal		↔	↔	↔	↓	↓	↔
Submaximal vs. supramaximal		↔	↔	↔	↓	↔	↔
INTERRATION TIME × CONDITION							
Control	PRE vs. POST	↔	↔	↔	↔	—	↔
	T_00_ vs. PRE	↔	↔	↔	↔	—	—
	T_00_ vs. T_05_	↔	↔	↔	↔	—	—
	T_10_ vs. T_00_	↔	↔	↔	↔	—	—
	T_15_ vs. T_00_	↔	↔	↔	↔	—	—
	POST vs. T_15_	↔	↔	↔	↔	—	—
Submaximal	PRE vs. POST	↔	↔	↓	↔	—	↓
	T_00_ vs. PRE	↓	↓	↑	↑	—	—
	T_00_ vs. T_05_	↔	↔	↔	↔	—	—
	T_10_ vs. T_00_	↑	↔	↔	↔	—	—
	T_15_ vs. T_00_	↑	↔	↔	↓	—	—
	POST vs. T_15_	↑	↑	↓	↓	—	—
Supramaximal	PRE vs. POST	↔	↔	↓	↔	—	↓
	T_00_ vs. PRE	↓	↓	↑	↑	—	—
	T_00_ vs. T_05_	↔	↔	↔	↑	—	—
	T_10_ vs. T_00_	↔	↔	↔	↓	—	—
	T_15_ vs. T_00_	↑	↑	↔	↓	—	—
	POST vs. T_15_	↑	↑	↓	↓	—	—

Abbreviations: − = no comparison performed in the statistical model or no significant main effect or interaction (*p* > 0.05), ↑ = Post hoc reported that first condition of the comparison were greater than second reported condition of the comparison (*p* < 0.05), ↓ = post hoc reported that first condition of the comparison were lesser than second reported condition of the comparison (*p* < 0.05), ↔ = post hoc reported that both conditions of the comparison were similar (*p* > 0.05), CON = control session, GM *H*_max_/*M*_max_ = gastrocnemius medial maximal *H*_reflex_ normalized by maximal *M*_wave_, MVC = maximal voluntary contraction, POST = posttest, PRE = pretest, PT = passive torque, PTT = peak twitch torque, ROM_max_ = maximal range of motion, SOL *H*_max_/*M*_max_ = soleus maximal *H*_reflex_ normalized by maximal *M*_wave_, Submaximal = static stretching submaximal, Supramaximal = static stretching supramaximal, T_00_ = measure immediately at the begin of the experimental condition, T_05_ = measure 5 min after the begin of the experimental condition, T_10_ = measure 10 min after the begin of the experimental condition, T_15_ = measure 15 min after the begin of the experimental condition.

For GM *H*_max_/*M*_max_, there was no significant effect of condition (χ^2^ = 5.59, *p* = 0.061). However, there was a significant main effect of time (χ^2^ = 93.94, *p* = 0.001) and a significant time × condition interaction (χ^2^ = 57.86, *p* = 0.001). GM *H*_max_/*M*_max_ at T_15_ was greater than at T_00_ only in the supramaximal condition (*p* = 0.008). Additionally, GM *H*_max_/*M*_max_ at T_00_, T_05_, T_10_, and T_15_ were significantly lower than at PRE and POST in both supramaximal (*p* < 0.01) and submaximal (*p* = 0.01) conditions. Conversely, no significant differences were found in GM *H*_max_/*M*_max_ between T_05_, T_10_, and T_15_ (*p* > 0.05), nor between PRE and POST in any experimental condition (*p* > 0.05). GM *H*_max_/*M*_max_ effect sizes between PRE and POST were 0.10 (trivial), 0.01 (trivial), and 0.12 (trivial) for control, submaximal, and supramaximal conditions, respectively. Additionally, GM *H*_max_/*M*_max_ effect sizes between T_00_ and T_15_ were −0.04 (trivial), 0.50 (small), and 1.06 (moderate) for control, submaximal, and supramaximal conditions, respectively.

**FIGURE 4:**
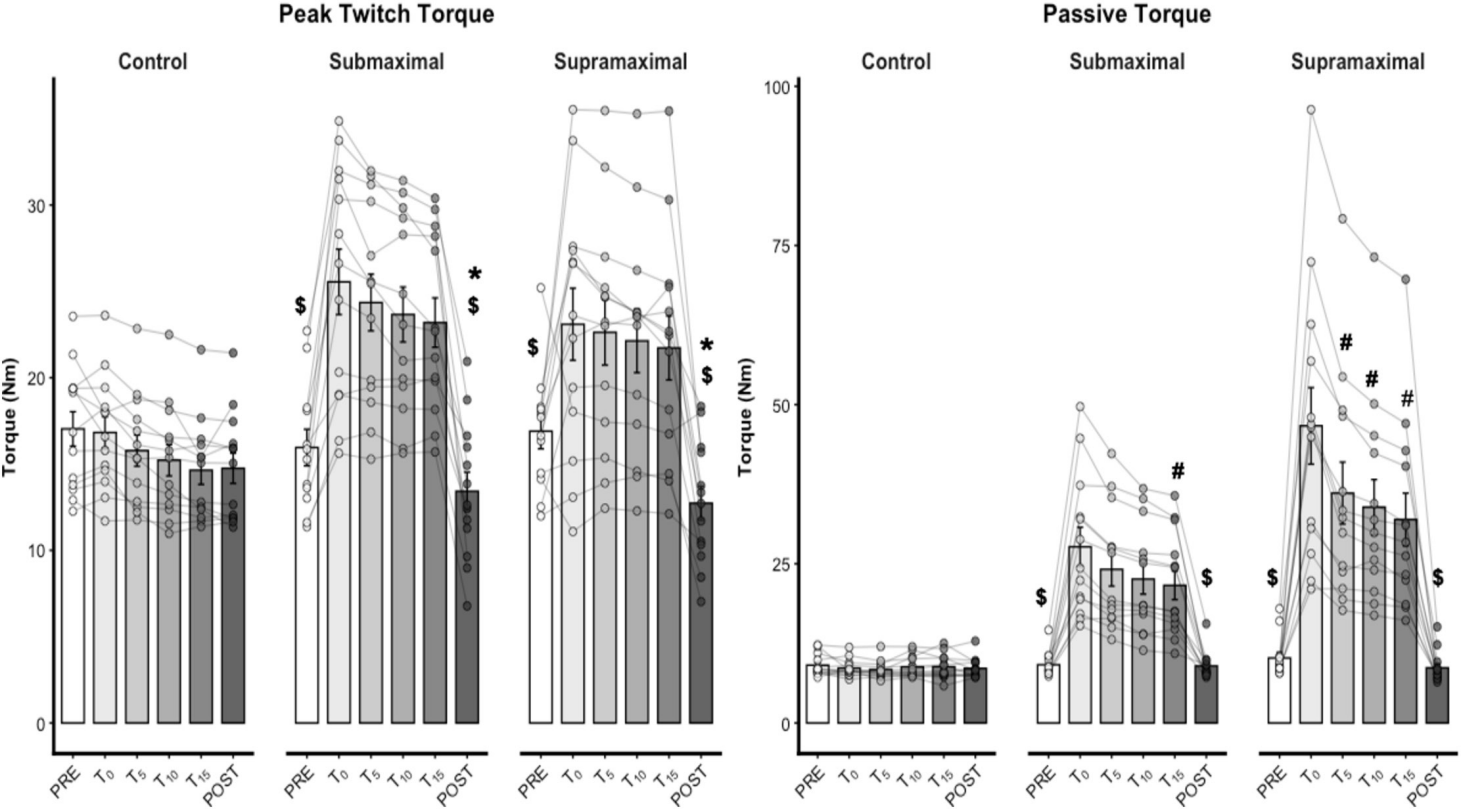
Mechanical adaptations (*n* = 12 participants). Control = control condition, ES _PREvs.POST_ = effect size between PRE and POST moments, ES _T00vs.T15_ = effect size between T_00_ and T_15_ moments, POST = posttest, PRE = pretest, PT = passive torque, PTT = peak twitch torque, Submaximal = static stretching submaximal condition, Supramaximal = static stretching supramaximal condition, T_00_ = measure immediately at the begin of the experimental condition, T_05_ = measure 5 min after the begin of the experimental condition, T_10_ = measure 10 min after the begin of the experimental condition, T_15_ = measure 15 min after the begin of the experimental condition. *Significant differences from PRE moment (*p* < 0.05). #Lesser than T_00_ moment (*p* < 0.05). ^$^Greater than both PRE and POST moments (*p* < 0.01).

For PTT, there was no significant effect of condition (χ^2^ = 4.77, *p* = 0.092). However, there were significant main effects of time (χ^2^ = 284.08, *p* = 0.001) and a significant time × condition interaction (χ^2^ = 194.39, *p* = 0.001). PTT at PRE was greater than POST in both supramaximal (*p* = 0.001) and submaximal (*p* = 0.006) conditions. Additionally, PTT at T_00_, T_05_, T_10_, and T_15_ was greater than PRE and POST in both supramaximal (*p* = 0.001) and submaximal (*p* = 0.001) conditions (Figure 4). Conversely, there were no significant differences for PTT at T_00_, T_05_, T_10_, and T_15_ in all experimental conditions (*p* > 0.05), nor between PRE and POST at the control condition (*p* = 0.054). PTT effect size between PRE and POST moments was −0.61 (small), −0.71 (small), and −1.19 (moderate) for control, submaximal, and supramaximal conditions, respectively. Additionally, PTT effect size between T_00_ and T_15_ moments was −0.67 (small), −0.35 (trivial), and −0.19 (trivial) for control, submaximal, and supramaximal conditions, respectively.

For PT, there were significant main effects of condition (χ^2^ = 1140.89, *p* = 0.001) and time (χ^2^ = 942.96, *p* = 0.001), as well as a significant time × condition interaction (χ^2^ = 537.49, *p* < 0.001). In the supramaximal condition, PT at T_00_ was lower than at T_05_, T_10_, and T_15_ (*p* < 0.001). Nonetheless, for the submaximal condition, PT at T_00_ was lower than only at T_15_ (*p* = 0.043). Additionally, T_00_, T_05_, T_10_, and T_15_ were significantly greater than PRE and POST in both supramaximal (*p* < 0.01) and submaximal (*p* = 0.01) conditions. Conversely, no significant differences were found in PT between T_05_, T_10_, and T_15_ (*p* > 0.05), nor between PRE and POST in any experimental condition (*p* > 0.05). PT effect sizes between PRE and POST were −0.30 (trivial), −0.10 (trivial), and −0.49 (small) for control, submaximal, and supramaximal conditions, respectively. Additionally, PT effect sizes between T_00_ and T_15_ were 0.15 (trivial), −0.55 (small), and −0.68 (small) for control, submaximal, and supramaximal conditions, respectively.

**FIGURE 5:**
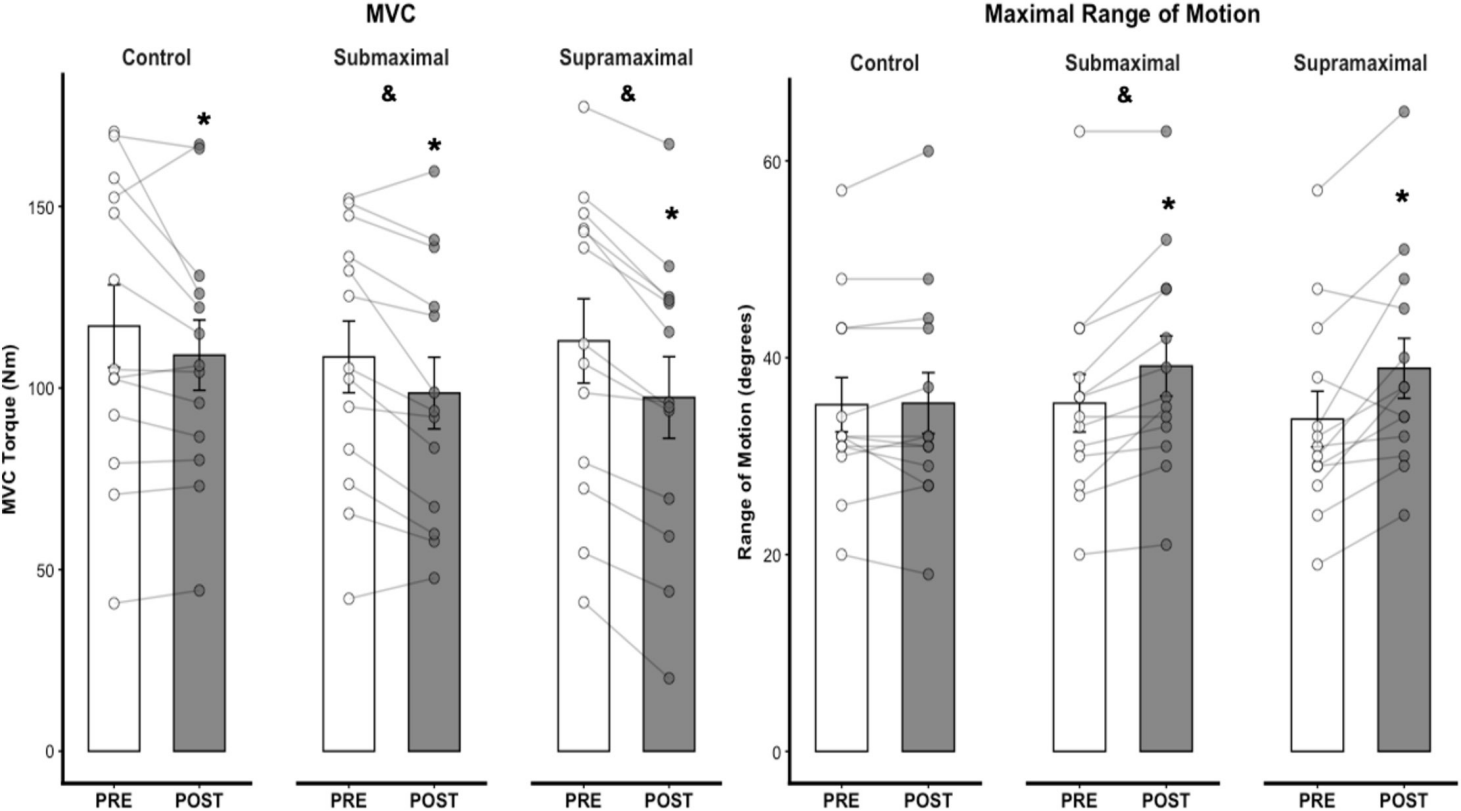
Muscle strength and flexibility adaptations (*n* = 13 participants). Control = control condition, MVC = maximal voluntary contraction, PRE = pretest, POST = posttest; Submaximal = submaximal static stretching condition, Supramaximal = supramaximal static stretching condition. *Significant differences from PRE moment (*p* < 0.05). &Main effect for condition with significant differences from control.

For MVC, there was no significant time × condition interaction (χ^2^ = 2.36, *p* = 0.307, Figure 5). However, there were significant main effects of time (χ^2^ = 12.61, *p* = 0.001) and condition (χ^2^ = 9.20, *p* = 0.010). MVC at POST was lower than PRE (*p* = 0.001). The MVC decrease was greater in both supramaximal (*p* = 0.016) and submaximal (*p* = 0.035) conditions when compared to the control condition. No differences were found between supramaximal and submaximal conditions (*p* = 0.959). MVC effect sizes between PRE and POST were −0.20 (trivial), −0.28 (trivial), and −0.37 (small) for control, submaximal, and supramaximal conditions, respectively.

For ROM_max_, there were significant main effects of time (χ^2^ = 27.18, *p* = 0.001), condition (χ^2^ = 8.69, *p* = 0.013), and time × condition interaction (χ^2^ = 15.89, *p* = 0.001). ROM_max_ at POST was greater than at PRE in both supramaximal (*p* = 0.001) and submaximal (*p* = 0.001) conditions. Conversely, there were no significant differences between PRE and POST ROM_max_ in the control condition (*p* = 1.000). ROM_max_ effect sizes between PRE and POST were 0.02 (trivial), −0.36 (small), and −0.50 (small) for control, submaximal, and supramaximal conditions, respectively.

## 4 Discussion

This study demonstrates that prolonged static stretching elicits biphasic modulation of spinal excitability, characterized by initial suppression followed by progressive facilitation that is amplitude‐dependent; supramaximal stretching elicits greater neural adaptations in both the SOL and gastrocnemius muscles. In contrast, submaximal stretching only affects the SOL. Concurrently, PT initially increased during stretch application but subsequently declined more rapidly under supramaximal conditions (5 min) compared to submaximal conditions (15 min), indicating differential viscoelastic adaptations within the muscle‐tendon unit. Despite these neural and mechanical differences, both stretching amplitudes similarly reduced voluntary muscle strength while improving joint ROM, and slightly greater flexibility gains are observed under supramaximal conditions.

The initial decrease in spinal excitability during static stretching aligns with previous findings reporting reduced *H*_max_ or *H*_max_/*M*_max_ during passive stretching maneuvers (Guissard et al. 2001; Budini et al. 2018). Since the reduction in reflex responses may contribute to greater muscle flexibility (Guissard and Duchateau 2006), it supports the notion that stretching may induce changes in both neural and peripheral inputs to the motoneuron pool, thereby facilitating muscle lengthening (Guissard et al. 2001; Guissard and Duchateau 2006). The amplitude *H*_max_/*M*_max_ is modulated by excitatory and inhibitory mechanisms in the α‐motor neurons (Clark et al. 2014; Stevanovic et al. 2019). For instance, a previous study measured SOL *H*_max_ every 3 s during a 30‐s static stretch of the calf muscles and reported a decrease at the onset of stretching. However, because of the recovery characteristics of *H*_max_ measurements, the responses showed an enhancement from 21 s onward compared with the first stimulus at 3 s. The authors attributed this early reduction in *H*_max_ to decreased neurotransmitter release, suggesting homosynaptic depression (Budini et al. 2018). Nevertheless, postactivation depression of the Hoffman reflex is typically observed when stimuli are delivered at short intervals because recovery of H‐reflex responses generally requires 8 s (Vitry et al. 2021). Therefore, in the absence of a control condition in the previous study (Budini et al. 2018), the observed depression may have resulted from the stimulation protocol itself rather than from the stretching intervention (Budini et al. 2018; Vitry et al. 2021). Thus, although evidence suggested that homosynaptic depression may play an important role in the modulation of spinal excitability during stretching (Budini et al. 2018; Datoussaid et al. 2021), the reduction in the *H*_max_/*M*_max_ at the onset of stretching observed in the present study cannot be unequivocally attributed to this mechanism, as it remains uncertain whether homosynaptic depression was specifically induced by the stretching exercise or by the stimulation protocol itself (Budini et al. 2018; Vitry et al. 2021). Accordingly, other neurophysiological mechanisms likely contributed to the attenuation of the reflex response.

Consistent with this interpretation, a previous study reported reductions in both the exteroceptive reflex (*E*_reflex_/*M*_max_) and the motor evoked potential (MEP/*M*_max_) in the calf muscles during ankle dorsiflexion at 20° compared to the neutral position (Guissard et al. 2001). Because spinal motor neurons integrate afferent inputs originating from both cortical and peripheral sources (Aagaard et al. 2002; Heckman et al. 2005), and *E*_reflex_/*M*_max_ and MEP/*M*_max_ are considered to reflect, respectively, afferent input from nociceptive nerve endings and corticospinal drive to the spinal motoneuron pool (Guissard et al. 2001; Pulverenti et al. 2020; Vieira et al. 2025), the initial decrease in spinal excitability during static stretching could result from concurrent reductions in both peripheral afferent feedback from nociceptive nerve endings and cortical excitatory input (Guissard et al. 2001; Guissard and Duchateau 2006). However, reductions in *H*_max_ amplitude during stretching induced by tendon pressure have been observed even after local anesthesia of the skin (Robinson et al. 1982), suggesting that nociceptive nerve endings' activity may not play a predominant role in spinal excitability modulation during muscle lengthening (Robinson et al. 1982; Masugi et al. 2017). Conversely, Ia afferent transmission from muscle spindles during muscle lengthening is suppressed by presynaptic inhibition (Guissard and Duchateau 2006), providing an additional inhibitory contribution to spinal motoneuron excitability. Collectively, these mechanisms suggest that static stretching transiently engages multiple inhibitory pathways to reduce motoneuron output (Guissard et al. 2001; Guissard and Duchateau 2006), and thereby, it could help explain the decline in *H*_max_/*M*_max_ observed at the onset of stretching in our study.

Despite an initial decline in spinal excitability at the onset of stretching, *H*_max_/*M*_max_ progressively increased throughout the static stretching period, independent of stretching magnitude. Notably, this facilitation was observed in both the SOL and medial gastrocnemius under supramaximal conditions, and it was limited to the SOL under submaximal conditions. A plausible explanation is that adaptations within the muscle–tendon unit contributed to the observed increase in spinal excitability. Consistent with this, our study reported that PT decreased across all stretching conditions, with a greater reduction during supramaximal stretching (effect size = 0.68; ~30% decrease from T_00_ to T_15_) compared with submaximal stretching (effect size = 0.55; ~22% decrease from T_00_ to T_15_). Previous studies have shown that static stretching promotes elongation at the myotendinous junction (Mizuno et al. 2012; Maeda et al. 2017). Importantly, such extensibility appears to originate primarily from adaptations in the muscle fibers rather than the tendon itself, as elongation of the calf muscles can occur without measurable displacement of the Achilles tendon following static stretching (Morse et al. 2008). Therefore, muscle elongation required to maintain the ankle joint at the target stretching angle may enhance muscle spindle activity, potentially increase afferent feedback, and contribute to the elevations in *H*_max_/*M*_max_ observed throughout the stretching (Blum et al. 2017; Kröger and Watkins 2021). In line with this interpretation, Datoussaid et al. (2021) reported that changes in the *H*_max_/*M*_max_ ratio during the loading and unloading phases of stretching were inversely related to PT, suggesting a mechanistic link between stretch‐induced reductions in muscle stiffness and modulation of spinal excitability.

However, the elevations in *H*_max_/*M*_max_ observed during stretching appear to be transient (Guissard et al. 2001; Budini et al. 2018, 2019). For example, previous studies have reported that a pronounced decrease in *H*_max_/*M*_max_ during passive stretching rapidly returned to baseline values once stretching ceased (Guissard et al. 2001; Budini et al. 2018). Likewise, other investigations demonstrated that although *H*_max_/*M*_max_ responses increased immediately after one or 5 min of static stretching, they reverted to baseline within 2 min (Budini et al. 2017, 2019). Despite the longer duration applied in our protocol (i.e., 15 min), our results were consistent with these previous findings across different stretching volumes (Budini et al. 2017, 2019). Taken together, this evidence suggests that the adaptations in *H*_max_/*M*_max_ responses observed during or immediately after static stretching are short‐lived and return to baseline within minutes following the cessation of stretching (Guissard et al. 2001; Budini et al. 2018).

In contrast to the *H*_max_/*M*_max_ responses, PT increased immediately at the onset of stretching, with a greater increase observed during the supramaximal compared with the submaximal stretching condition. These findings are consistent with previous studies reporting higher PT at greater ankle dorsiflexion ranges of motion during muscle lengthening (Mizuno and Umemura 2016; Mizuno 2017). Despite the initial increase in PT at the onset of stretching, PT progressively decreased throughout the stretching period, with slightly greater reductions in the supramaximal than in the submaximal condition (effect sizes of −0.68 vs. −0.55 for supramaximal and submaximal stretching conditions, respectively). Previous evidence suggested that decreases in PT may result from a reduction in afferent input from nociceptive nerve endings and mechanoreceptors (Guissard et al. 2001; Guissard and Duchateau 2006; Lévenéz et al. 2023). As the activity of both nociceptive afferents and mechanoreceptors decreases during muscle lengthening, particularly at higher ankle dorsiflexion ranges of motion, such changes could, in principle, reduce spinal excitability and decrease PT (Guissard et al. 2001). However, in the present study, spinal excitability increased throughout the stretching protocol, as evidenced by a higher *H*_max_/*M*_max_ ratio at the end compared to the onset of stretching. This dissociation suggests that the observed changes in PT were unlikely to be mediated by alterations in spinal excitability associated with nociceptive or mechanoreceptive afferent input. Accordingly, the reduction in PT observed during stretching is more likely attributable to changes in the stiffness of the muscle and surrounding connective tissues within the muscle–tendon unit (Opplert et al. 2016; Opplert and Babault 2019; Vieira et al. 2021; Lévenéz et al. 2023). In this context, greater static stretching amplitudes are known to induce larger reductions in muscle–tendon stiffness (Takeuchi and Nakamura 2020; Takeuchi, Akizuki, and Nakamura 2021), and therefore, it could help to explain the slightly greater decrease in PT throughout the stretching period, observed under the supramaximal compared with the submaximal stretching condition.

Similarly, both protocols increased joint ROM, with slightly greater improvements following supramaximal compared with submaximal stretching (effect sizes of 0.50 vs. 0.35, respectively), which is consistent with previous studies reporting flexibility gains after stretching performed at high or moderate amplitudes (Takeuchi, Akizuki, and Nakamura 2021; Marchetti et al. 2022). Enhancements in ROM are commonly attributed to reductions in muscle–tendon viscoelasticity (Bouvier et al. 2017). Elastography studies have shown decreases in shear‐wave velocity following static stretching (Morrin and Redding 2013; Reiner et al. 2022), and other investigations using stiffness indices have also reported reductions in muscle–tendon stiffness (Nojiri et al. 2019; Palmer et al. 2022). Our study observed reductions in PT under both conditions, with slightly greater decreases after supramaximal than submaximal stretching (effect sizes of 0.68 and 0.55, respectively, between the T_00_ and T_15_ measures). Moreover, although evoked contractile properties (i.e., peak twitch torque) increased during the stretching protocol, possibly due to the length–tension relationship (Maffiuletti and Lepers 2003), greater impairments in evoked contractile properties were observed after supramaximal compared with submaximal stretching (effect size of −1.19 and −0,71, respectively). PT is often considered a surrogate marker of muscle–tendon stiffness, and evoked contractile properties, though primarily reflecting contractile capacity, may also indicate changes in muscle and tendon stiffness (Ryan et al. 2011; Vieira et al. 2021). Therefore, the combined reductions in PT and peak twitch torque suggest that stretching decreased muscle–tendon stiffness, particularly when performed at greater amplitudes. Consequently, given the established relationship between muscle–tendon stiffness and ROM (Bouvier et al. 2017), the greater reductions in these stiffness‐related markers following stretching observed in our study, especially after the greater stretching amplitudes, may help explain the slightly greater improvements in ROM observed in this condition compared with the submaximal condition.

In addition, our results demonstrated a similar reduction in voluntary muscle strength following both supramaximal and submaximal static stretching compared with the control condition. Previous systematic review indicates that greater impairments in strength may occur after short‐duration static stretching (e.g., 2 × 30 s) when performed at maximal rather than submaximal amplitudes. However, when stretching volume is higher, different amplitudes may induce similar deficits (Bryant et al. 2023). The physiological mechanisms underlying these impairments remain incompletely understood, but intrinsic motoneuron properties, particularly persistent inward currents (PICs), are thought to play a key role. PICs amplify synaptic input and enhance the input–output relationship of motoneurons, thereby supporting sustained force production during voluntary contractions, such as those assessed in our study (Heckman et al. 2005). Prolonged static stretching has been shown to reduce PIC activity (Trajano and Blazevich 2021), which provides a plausible explanation for the attenuation of voluntary strength observed in the present findings. Although we observed slightly greater impairments in evoked contractile properties following supramaximal compared with submaximal stretching, possibly reflecting disturbances in cross‐bridge function, prior evidence suggests that static stretching–induced reductions in muscle strength are primarily of neural rather than peripheral origin (Trajano et al. 2013). This may explain the absence of significant voluntary strength differences between conditions despite divergent evoked contractile responses.

Our study has some limitations that should be acknowledged. The *H*_max_/*M*_max_, PTT, and PT measures during the experimental protocols (T_00_ to T_15_) were obtained with the ankle positioned differently across conditions (i.e., ROM_max_ + 5°, ROM_max_ − 5°, or neutral), which could have an impact on the results (Guissard et al. 2001; Vieira et al. 2025). In addition, during the stretching sessions, PRE and POST measures were assessed in the neutral position, whereas measures obtained during stretching were performed at ROM_max_ + 5° or ROM_max_ − 5°. Although these methodological discrepancies may introduce variability, the generalized mixed model provides a robust statistical framework accounting for interindividual variability (Cnaan et al. 1997), and covariate inclusion was intended to minimize type I error and enhance analytical conservatism (Hernández et al. 2006). Moreover, the *H*_max_ and *M*_max_ intensities were determined from the recruitment curve at the beginning of each experimental session. Therefore, intensities were the same for PRE, T00, T05, T10, T15, and POST moments. Additionally, performing a recruitment curve at each time point to measure *H*_max_ and *M*_max_ would be time‐consuming and therefore mask potential immediate and transient stretch effects (Budini et al. 2017; Opplert et al. 2020; Vieira et al. 2025). Indeed, the use of a constant intensity throughout an experimental session is usually conducted in the literature on the acute effect of stretching on the *H*_max_/*M*_max_ ratio (Opplert et al. 2020; Vieira et al. 2025).

## 5 Conclusion

Static stretching amplitude plays a key role in modulating neuromechanical adaptations. Both supramaximal and submaximal stretching improved joint flexibility, likely through reductions in muscle–tendon stiffness, with slightly greater ROM gains observed under supramaximal conditions. Spinal excitability initially decreased but subsequently increased to a greater extent during supramaximal stretching compared with submaximal protocols. Nevertheless, both conditions elicited similar decreases in voluntary muscle strength. These findings highlight the importance of considering stretching amplitude when designing flexibility or rehabilitation programs. By demonstrating that higher amplitudes can acutely enhance flexibility without inducing additional strength impairments, this study provides novel insights into the neural and mechanical mechanisms underlying static stretching. Additionally, it informs safer and more effective stretching programs to be applied in sports and clinical contexts.

## Author Contributions

Conception and design of the work: D.C.L.V. and N.B. Acquisition, analysis, or interpretation of the data for the work: D.C.L.V., M.H., J.L.Q.D., N.B., and M.B. Drafting the work or revising it critically for important intellectual content: all authors. All authors have read and approved the final version of this manuscript and agree to be accountable for all aspects of the work in ensuring that questions related to the accuracy or integrity of any part of the work are appropriately investigated and resolved. All persons designated as authors qualify for authorship, and all those who qualify for authorship are listed.

## Funding

The study was supported by the Centre d'Expertise de la Performance from the Université́ of Bourgogne, by the Region Bourgogne Franche‐Comté (2020Y‐22065 and 2022Y‐13186), by the National Council for Scientific and Technological Development–CNPq (304136/2020‐4, 200391/2022‐4, and 446576/2024‐7), Coordination for the Improvement of High Education Personnel (88887.188974/2025‐00), and by the Foundation for Support of Research in the Federal District‐FAP/DF (00193‐00001261/2021‐23, 00193‐00001661/2024‐81, and 00193‐00001372/2024‐82).

## Ethics Statement

The study received approval from the Ethics Committee for Research in STAPS (CERSTAPS IRB00012476‐2023‐17‐02‐229).

## Consent

All individuals were fully informed about the experimental procedure and purpose of the study, and they read and signed an informed consent form. The study followed the standards set out by the *Declaration of Helsinki*.

## Conflicts of Interest

The authors declare no conflicts of interest.

## Data Availability

Source data for this study are openly available at: https://github.com/denisclvieira/STRETCHAMPLITUDE.git.
